# Supplementary material for: Spin-decoupling of vertical cavity surface-emitting lasers with complete phase modulation using on-chip integrated Jones matrix metasurfaces
Source: Nat Commun. 2022 Dec 17;13:7795. doi: 10.1038/s41467-022-34977-0 (PMC9759547; doi:10.1038/s41467-022-34977-0)
Supplement: Supplementary file 1 — Supplementary Information [file 41467_2022_34977_MOESM1_ESM.pdf]

Supplementary Information

Spin-decoupling of Vertical Cavity Surface-Emitting Lasers with complete phase modulation using on-chip integrated Jones matrix metasurfaces

Pei-Nan Ni[1], Pan Fu[2], Pei-Pei Chen[3,*], Chen Xu[2], Yi-Yang Xie[2,*], and Patrice Genevet[1,*]

[1] Université Côte d'Azur, CNRS, Centre de Recherche sur l'Hétéro-Epitaxie et ses Applications (CRHEA), Valbonne, 06560, France

[2] Key Laboratory of Optoelectronics Technology, Beijing University of Technology, Ministry of Education, Beijing 100124, China

[3] Nanofabrication Laboratory, CAS Key Laboratory of Nanophotonic Materials and Devices, National Center for Nanoscience and Technology, Beijing 100190, China.

*correspondence to: chenpp@nanoctr.cn, xieyiyang@bjut.edu.cn, patrice.genevet@crhea.cnrs.fr

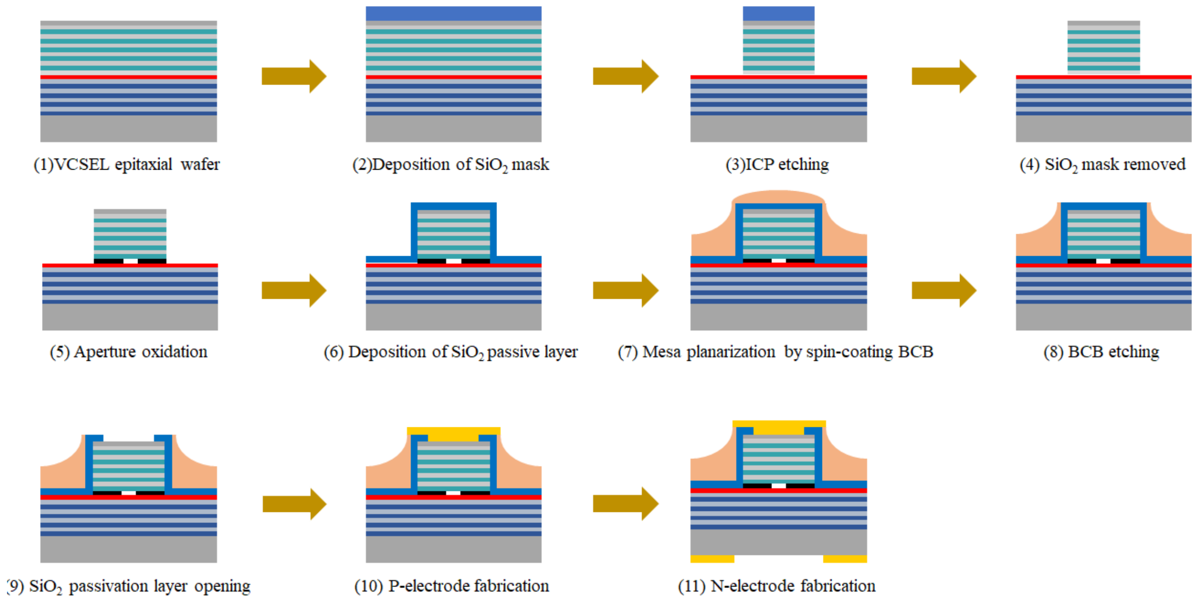

Supplementary Figure S1: Fabrication of back-side emitting VCSELs. (1) A large number of distributed Bragg reflectors (DBRs) composed of 30.5 pairs of *p*-type top DBRs and 28.5 pairs of *n*-type bottom DBRs are employed in the design of the laser structure so that the VCSELs can be operated under low injection currents without additional cooling. A 30 nm $Al_{0.98}Ga_{0.02}As$ oxidation layer is sandwiched between the top DBRs and the active region for optical and electrical confiments. **(2)** For the VCSEL processing, a 500 nm thick SiO_2 was first deposited on the top of epitaxy wafer as a hard mask by plasma enhanced chemical vapor deposition (PECVD). **(3)** After that, circular shaped mesas are defined into a diameter of 50 µm and height of 5 µm, using UV lithography and inductively coupled plasma reaction ion etching (ICP-RIE). **(4)** Then, the SiO_2 was further removed by BOE etching. **(5)** Then, a small size (~3µm in diameter) of oxide aperture was obtained by selective oxidation of the $Al_{0.98}Ga_{0.02}As$ layer, which plays a critical role in ensuring the single fundamental mode operation of the laser. **(6)** After that, another thick SiO_2 (500 nm) layer was deposited onto the top surface for passivation. **(7)** Then, the surface was planarized by spin-coating Benzocyclobutene (BCB). After that, the BCB layer **(8)** and the SiO_2 passivation layer **(9)** were removed by reactive ion etching (RIE) using SF_6, and by BOE chemical etching, respectively. Finally, top *P*-contacts **(10)** and bottom *N*-contacts **(11)** were defined by a double-side photolithography, lift-off and a rapid thermal annealing of Ti/Au and AuGeNi/Au, respectively, to ensure good ohmic contacts.

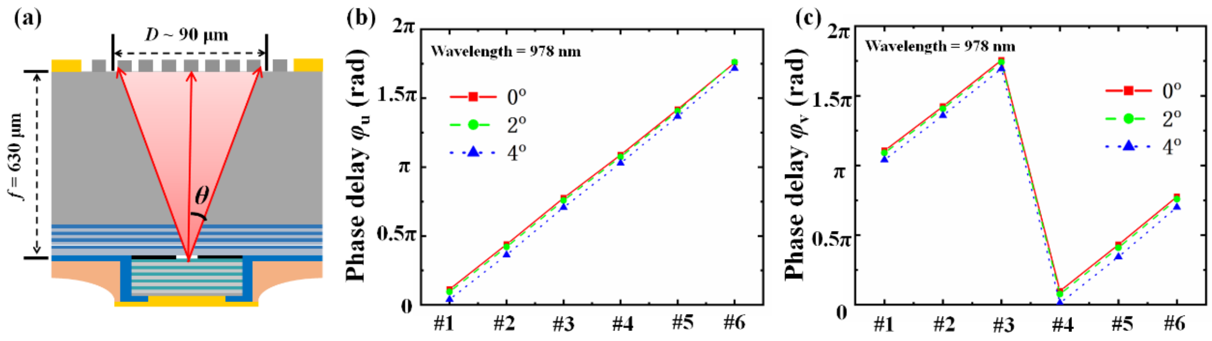

Supplementary Figure S2: The dependance of the meta-atom phase response on the incident angle. **(a)** Schematic illustration of the angle dependent incident beams upon the meta-atoms; Simulated propagation phase delay φ_u **(b)** and φ_v **(c)** of the selected six meta-atoms at a laser emission wavelength of 978 nm for different incident angles, which only exhibit small variations within this small range of incident angles.

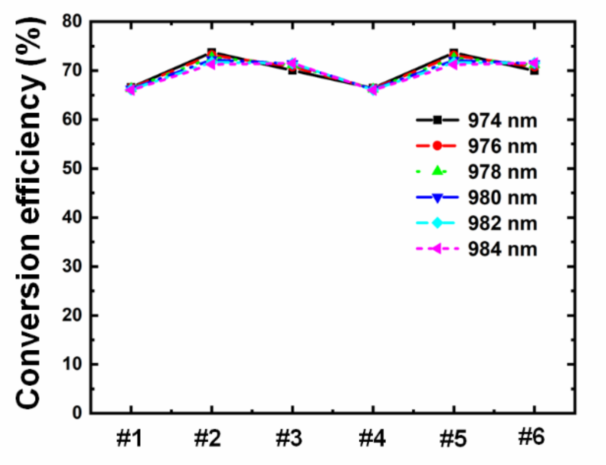

Supplementary Figure S3: Simulated conversion efficiency of the meta-atoms within the wavelength range of interest from 974 nm to 984 nm. Six rectangular nanopillars with a fixed heigh of 750 nm are selected as the meta-atoms in this work, of which the conversion efficiency is in the range of 65-75%. The dimensions of element 1-6 are #1: *W* = 123 nm, *L* = 235 nm; #2: *W* = 142 nm, *L* = 241 nm; #3: *W* = 155 nm, *L* = 253 nm; #4: *W* = 234 nm, *L* = 121 nm; #5: *W* = 241 nm, *L* = 141 nm; #6: *W* = 253 nm, *L* = 155 nm.

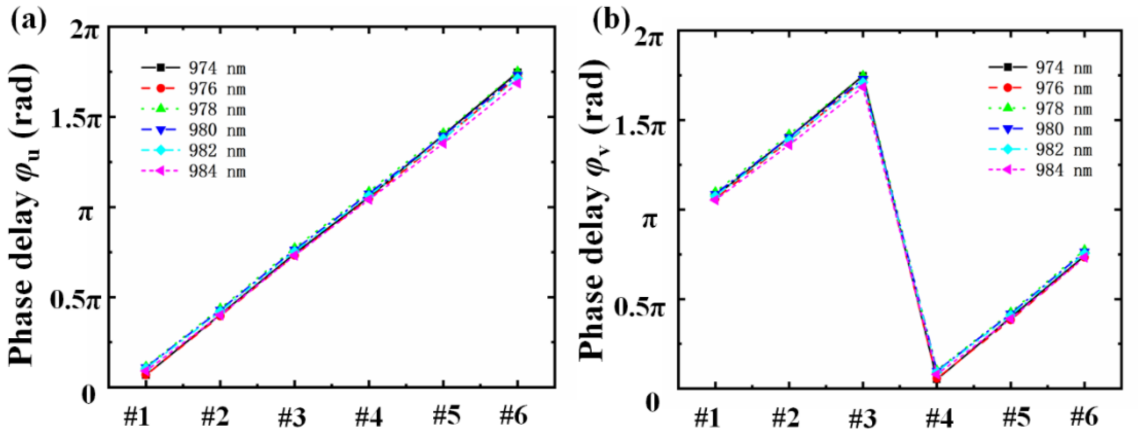

Supplementary Figure S4: Simulated propagation phase delay of the selected six meta-atoms within the wavelength range of interest from 974 nm to 984 nm. It shows that the propagation phases φ_u **(a)** and φ_v **(b)** of the meta-atoms barely change thanks to the large and relatively constant index of GaAs in this spectra range. Moreover, the intervals of the phase steps provided by those 6 elements remains the same in this wavelength region, keeping the phase discretization conditions unchanged.

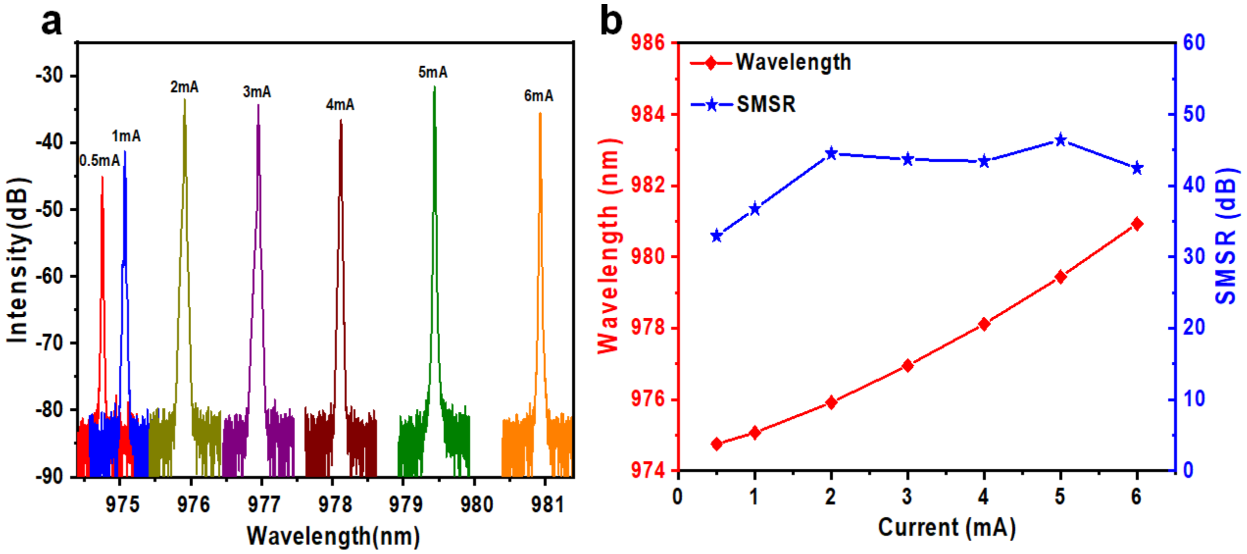

Supplementary Figure S5: The laser emitting characteristics of the fabricated VCSEL. (a) The emission spectral measured at different inject currents confirm the single-mode operation of the VCSEL up to the injection current of 6 mA. The resolution of our spectrometer is 0.01 nm. **(b)** shows the change of laser peak wavelength and the SMSR value as a function of the injected current, which reveals a weak dependance of laser wavelength on the injection current.

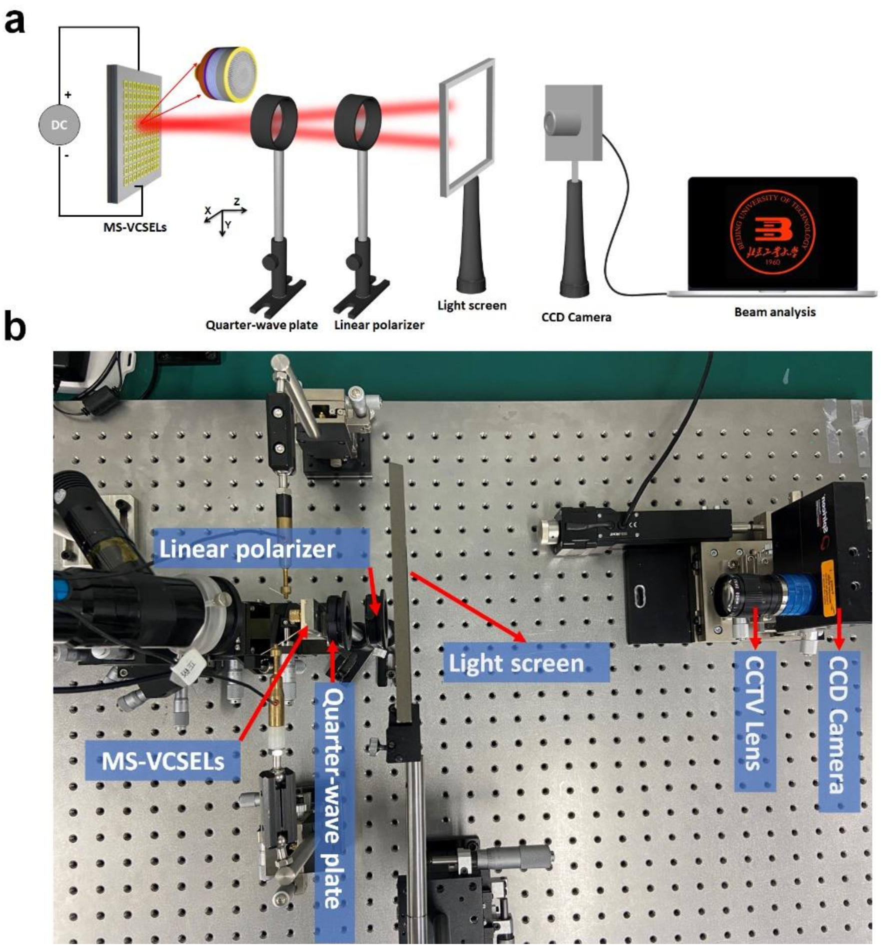

Supplementary Figure S6: Beam profiles measurement. The schematic (**a**) and experimental (**b**) setup for the far-field characterization of the beam intensity profiles of the spin-decoupled VCSELs. Quarter-wave plate in combined with a polarizer were employed to select the spin state of the VCSEL, which was projected onto a white screen for observation. An infrared CCD camera equipped with an objective (40× magnification, NA = 0.6) was used to record the intensity distribution of the laser beams.

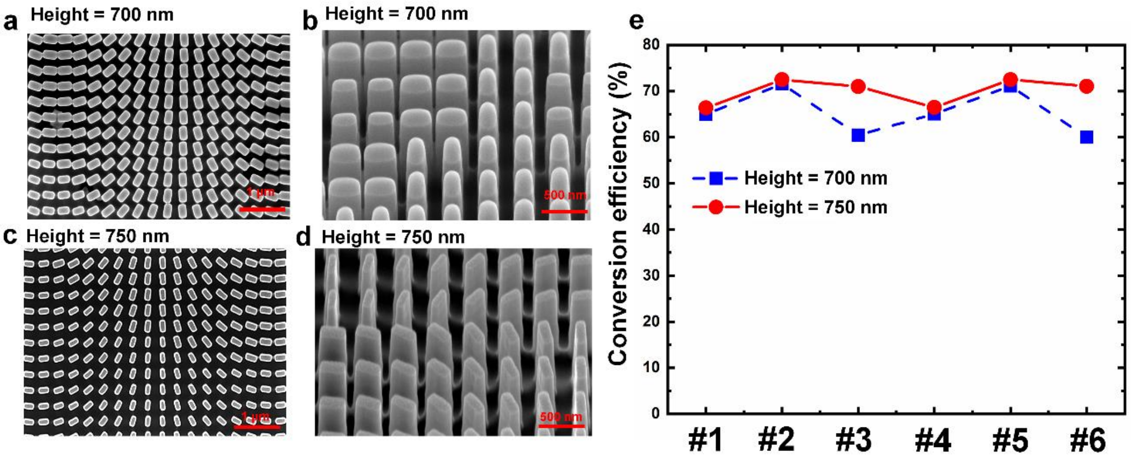

Supplementary Figure S7: The selected two sets of meta-atoms to construct metasurfaces with different conversion efficiency. Increasing the height of the meta-atoms from 700 nm to 750 nm allows us to select meta-atoms with smaller dimensions, as shown in **(a-d)**, to achieve the required phase modulation coverage while keeping the same lattice constant. It is worth noting that although the meta-atoms with different height only exhibit slight difference in conversion efficiency according to **(e)**, the larger dimensions of the 700-nm-tall meta-atoms caused more significant fabrication errors, mainly manifesting as larger variations of the etching depth among the nano-pillars with varying gaps, as revealed in **(b)**. This will cause substantial deviations of the device actual performance from the design, due to both the phase and transmission errors, especially for those meta-atoms with smaller separation distance. As a result, the fabricated metasurfaces with 700 nm height present a much stronger co-polarization beam spot, as observed in both the device B and device C. On the other hand, to further increase the performance of metasurface for highly efficient operations, precise control of the etching depth of meta-atoms with different dimensions can be achieved by introducing an etching stopping layer on the backside surface of the substrate. Then, an additional layer of GaAs with well-defined thickness was grown, which can provide ultrahigh resolution even in the sub-nanometer scale by taking the advantage of the epitaxy technology. Moreover, the overall performance of the device can be further enhanced by employing more phase levels to construct the metasurface to mitigate the substantial phase errors due to the relatively small number of phase discretization levels ($N = 6$) in the current design.

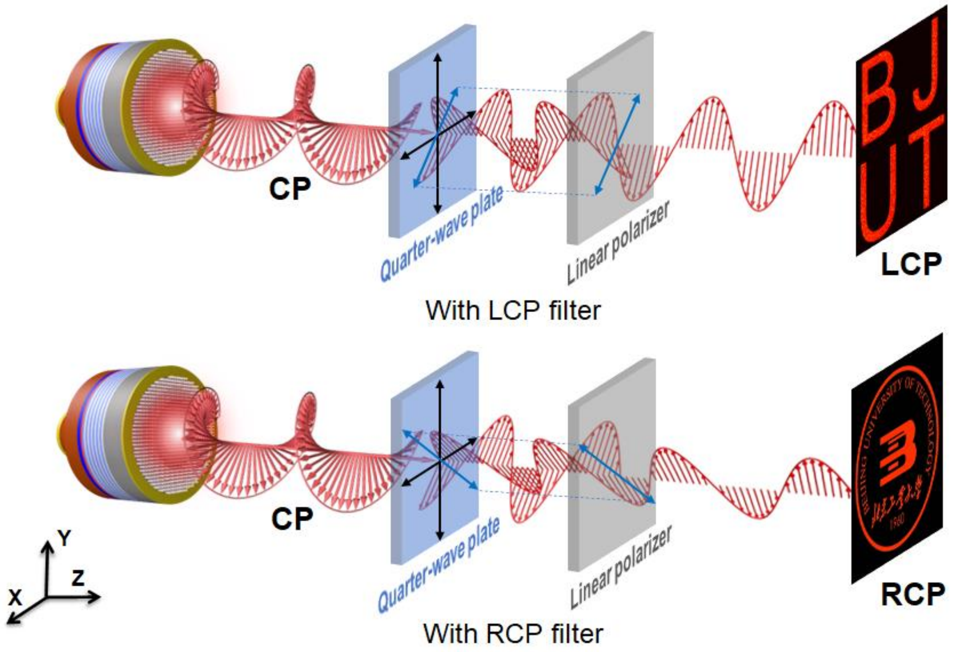

Supplementary Figure S8: Circular polarization state dependent measurements of the obtained dual-channel holographic display. The individual spin state channel carrying the holographic information can be selectively concealed in combination of the quarter-wave plate and a polarizer as illustrated in the above setup.

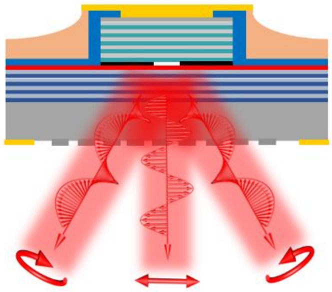

	RCP	LP	LCP
Propagation phase	$\phi_{collimator}$	$\phi_{collimator}$	$\phi_{collimator}$
PB phase	$\phi_{deflector}$	$None$	$-\phi_{deflector}$
Total Phase	$\phi_{collimator}$ $+\phi_{deflector}$	$\phi_{collimator}$	$\phi_{collimator}$ $-\phi_{deflector}$

$$\phi_{collimator} = 2\pi - \frac{2\pi n}{\lambda}\left(\sqrt{x^2 + y^2 + f^2} - f\right)$$

$$\phi_{deflector} = 2\pi - \frac{2\pi}{\lambda} y \cdot sin\alpha$$

Supplementary Figure S9: The resulting phase profiles for the generations of three directional beams with specified polarization states. In this example, a gradient phase profile $\phi_{deflector}$ is imposed to the RCP component using PB phase to deflect the RCP, while the LCP component will automatically gain an equal and opposite phase gradient. In addition, a hyperboloidal phase distribution: $\phi_{collimator}$ was introduced using propagation phase, which can compensate for the diffraction of both the cross-polarized and co-polarized components for collimating emissions.

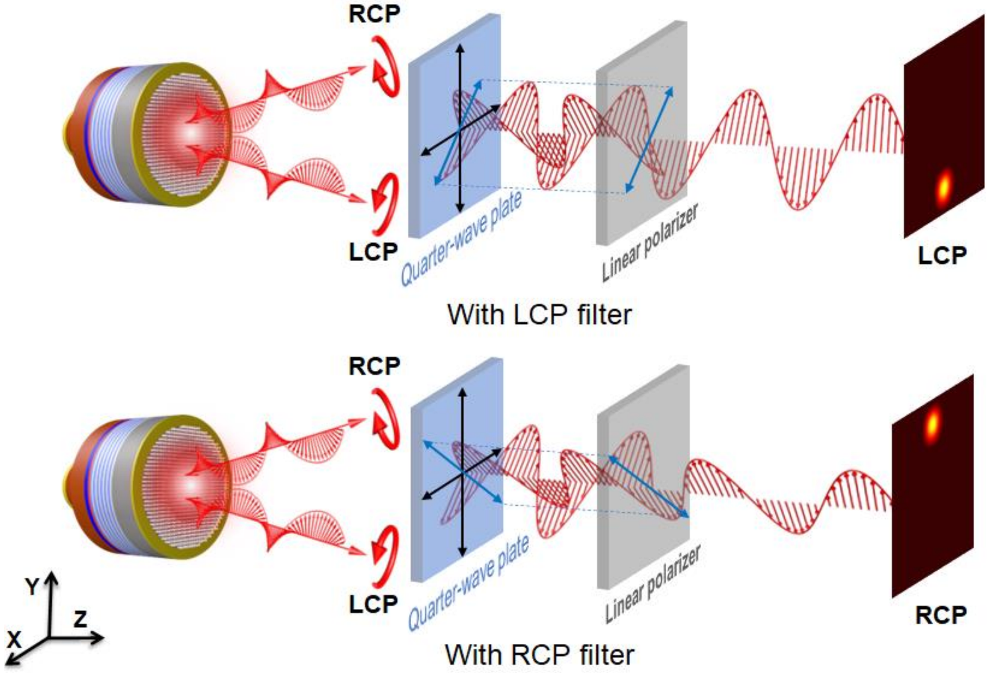

Supplementary Figure S10: The experimental setup for the polarization states characterization of the deflected beams emitted by the spin decoupled VCSEL. The individual spin state of the deflected beams can be determined in combination of the quarter-wave plate and a polarizer as depicted in the above setup.

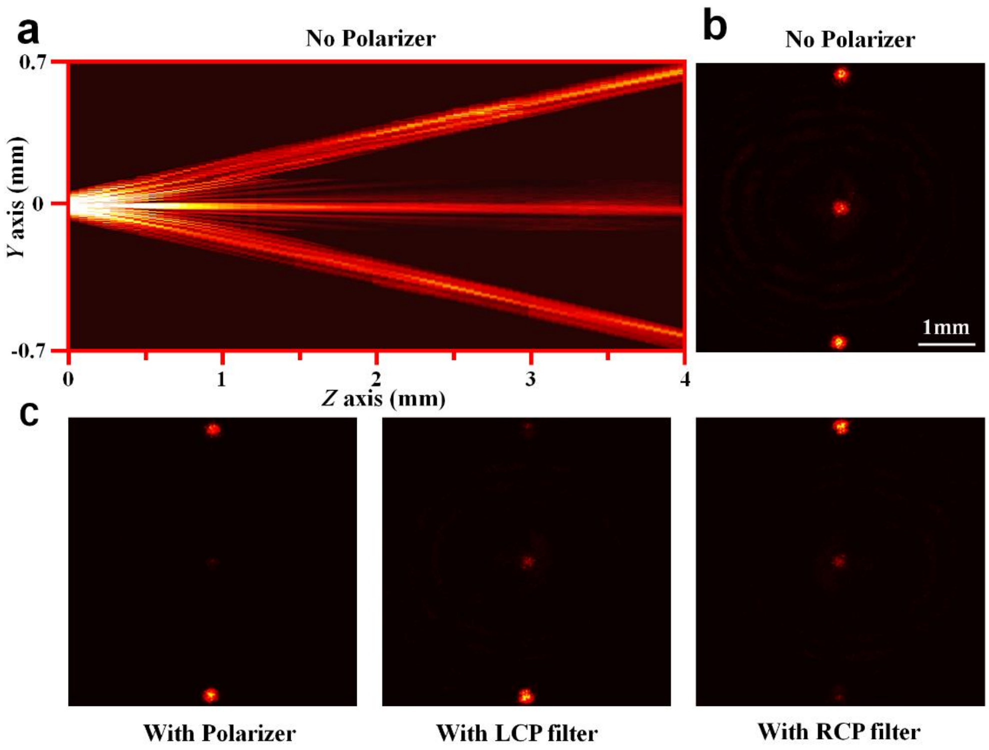

Supplementary Figure S11: Generation of interference-free multi-polarization channels. In this example, the metasurface (700 nm in height) that shows a smaller conversion efficiency than the 750-nm-tall metasurface was integrated with the VCSEL to further confirm the effectiveness of the design strategy under the existence of a stronger co-polarization beam. To this end, the beam intensity distributions of the VCSEL were presented both along *Z* direction up to 4 mm **(a)**, and at $Z = 1.5$ cm **(b)** without polarizer, respectively. And the SOPs of the generated beams were further determined using polarization analyzers **(c)**. The results show that two CP beams and one strong LP beam were collimated and well separated along different directions without noticeable interferences.

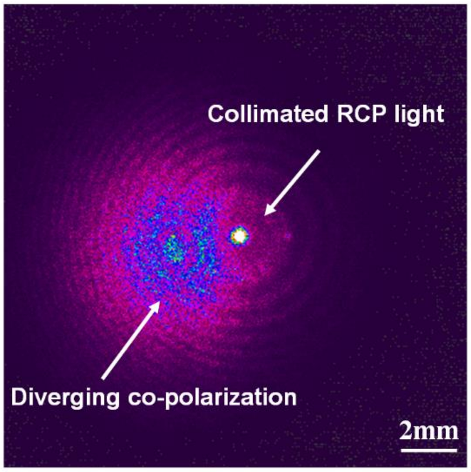

Supplementary Figure S12: Far-field beam profile of the integrated VCSEL using PB phase modulation alone measured at *Z* = 1.5 cm. In this design, both the collimation phase and the deflection phase are imposed to the RCP component of VCSEL by PB phase alone. Since the LCP beam will obtain a diverging phase due to the conjugate relationship with RCP light, it is hardly observed in the far filed measurement. In contrast to the well collimated RCP component, the co-polarization light remains highly divergent due to its unmodulated diverging phase profiles.
