# Supplementary material for: Spin-decoupling of vertical cavity surface-emitting lasers with complete phase modulation using on-chip integrated Jones matrix metasurfaces
Source: Nat Commun. 2022 Dec 17;13:7795. doi: 10.1038/s41467-022-34977-0 (PMC9759547; doi:10.1038/s41467-022-34977-0)
Supplement: Supplementary file 3 — Reporting Summary [file 41467_2022_34977_MOESM3_ESM.pdf]

nature research

Corresponding author(s): Pei-Pei Chen, Yi-Yang Xie, Patrice Genevet

Lasing Reporting Summary

Nature Research wishes to improve the reproducibility of the work that we publish. This form is intended for publication with all accepted papers reporting claims of lasing and provides structure for consistency and transparency in reporting. Some list items might not apply to an individual manuscript, but all fields must be completed for clarity.

For further information on Nature Research policies, including our data availability policy, see Authors & Referees.

ü Experimental design

Please check: are the following details reported in the manuscript?

1. Threshold

Plots of device output power versus pump power over a wide range of values indicating a clear threshold

☐ Yes
☒ No

Since this work studies beam shaping of commercially available VCSELs operated far above the threshold rather than a new report of lasing per se, we focused on the performance of lasers above their threshold. So we feel that the plots of output power vs pump power are not directly relevant to this study. We have of course measured the output power vs pumping power curve (P-I plot). We can provide this data on request.

2. Linewidth narrowing

Plots of spectral power density for the emission at pump powers below, around, and above the lasing threshold, indicating a clear linewidth narrowing at threshold

☐ Yes
☒ No

Laser spectra measured under different injection currents are shown both in Fig. 1b and Fig. S5a. And the linewidth of the laser spectrum above the threshold was discussed in the legend of Fig. 1, which confirmed the single fundamental mode operation of the VCSELs. Although the linewidth narrowing behavior is not directly relevant to our study, we can provide it on request.

Resolution of the spectrometer used to make spectral measurements

☒ Yes
☐ No

The resolution of our spectrometer for spectral measurements is given in the legend of Fig. S5.

3. Coherent emission

Measurements of the coherence and/or polarization of the emission

☒ Yes
☐ No

Measurements of the polarization of the emissions, which confirm well-defined polarization states of the far-field beams, are shown in Fig. 2, Fig. 3, Fig.4 and Fig.5, respectively.

4. Beam spatial profile

Image and/or measurement of the spatial shape and profile of the emission, showing a well-defined beam above threshold

☒ Yes
☐ No

Well-defined beam profiles of the devices above threshold are shown in Fig. 2, Fig.3, Fig.4 and Fig.5, respectively.

5. Operating conditions

Description of the laser and pumping conditions
Continuous-wave, pulsed, temperature of operation

☒ Yes
☐ No

The operating conditions of the lasers can be found in the legend of Fig.1.

Threshold values provided as density values (e.g. W cm^{-2} or J cm^{-2}) taking into account the area of the device

☐ Yes
☒ No

Although the threshold value is not relevant to this study, we can provide it on request.

6. Alternative explanations

Reasoning as to why alternative explanations have been ruled out as responsible for the emission characteristics
e.g. amplified spontaneous, directional scattering; modification of fluorescence spectrum by the cavity

☐ Yes
☒ No

The single mode laser spectrum in Fig. 1, and well-defined far-field beam profiles in Fig. 2, Fig. 3, Fig. 4 and Fig. 5 can directly confirm the lasing emissions of our VCSELs. Therefore, this is not a necessary consideration in this study.

7. Theoretical analysis

Theoretical analysis that ensures that the experimental values measured are realistic and reasonable
e.g. laser threshold, linewidth, cavity gain-loss, efficiency

☒ Yes
☐ No

Measurement results are compared with equation 2, equation 3, and equation 4, respectively, which show good agreement.

8. Statistics

Number of devices fabricated and tested

☒ Yes
☐ No

The number of the devices can be seen from Fig. 2, Fig. 3, Fig. 4, Fig. 5, Fig. S11 and Fig. S12, respectively.

Statistical analysis of the device performance and lifetime (time to failure)

☐ Yes
☒ No

Since the devices are fabricated from commercial laser wafer, we did not consider it necessary to perform statistical analysis of the device performance and lifetime. However, we note that no performance degradation was observed during the entire characterization of the devices.
